# Microgravity induces inhibition of osteoblastic differentiation and mineralization through abrogating primary cilia

**DOI:** 10.1038/s41598-017-02049-9

**Published:** 2017-05-12

**Authors:** Wengui Shi, Yanfang Xie, Jinpeng He, Jian Zhou, Yuhai Gao, Wenjun Wei, Nan Ding, Huiping Ma, Cory J. Xian, Keming Chen, Jufang Wang

**Affiliations:** 1 Gansu Key laboratory of Space Radiobiology, Institute of Modern Physics, Chinese Academy of Sciences, Lanzhou, 730000 P. R. China; 2 University of Chinese Academy of Sciences, Beijing, 100049 P. R. China; 3 Institute of Orthopaedics, Lanzhou General Hospital, Lanzhou Command of CPLA, Lanzhou, 730050 P. R. China; 4 Sansom Institute for Health Research, School of Pharmacy and Medical Sciences, University of South Australia, Adelaide, SA 5001 Australia

## Abstract

It is well documented that microgravity in space environment leads to bone loss in astronauts. These physiological changes have also been validated by human and animal studies and modeled in cell-based analogs. However, the underlying mechanisms are elusive. In the current study, we identified a novel phenomenon that primary cilia (key sensors and functioning organelles) of rat calvarial osteoblasts (ROBs) gradually shrank and disappeared almost completely after exposure to simulated microgravity generated by a random positioning machine (RPM). Along with the abrogation of primary cilia, the differentiation, maturation and mineralization of ROBs were inhibited. We also found that the disappearance of primary cilia was prevented by treating ROBs with cytochalasin D, but not with LiCl or dynein light chain Tctex-type 1 (Dynlt1) siRNA. The repression of the differentiation, maturation and mineralization of ROBs was effectively offset by cytochalasin D treatment in microgravity conditions. Blocking ciliogenesis using intraflagellar transport protein 88 (IFT88) siRNA knockdown inhibited the ability of cytochalasin D to counteract this reduction of osteogenesis. These results indicate that the abrogation of primary cilia may be responsible for the microgravity’s inhibition on osteogenesis. Reconstruction of primary cilia may become a potential strategy against bone loss induced by microgravity.

## Introduction

Along with the growing number of flights in space, more and more astronauts will be exposed to a harsh environment. While modern spacecrafts are designed to be effective barriers against many of the detrimental conditions such as extreme temperature and ionizing radiation, exposure to microgravity remains an unresolved issue^1^. Microgravity causes various problems in different biological systems including atrophy of the muscular system, dysfunction of the immune system, electrolyte imbalance and cardiovascular anomalies^2–4^. One of the most prominent and well recognized physiological challenges accompanying an extended spaceflight is the reduction in bone mass and osteopenia^5^.

Bone loss during spaceflights has been reproduced on the ground by microgravity-based research both *in vivo* and *in vitro*. One ground analog to microgravity is bed rest study, in which subjects remain in bed at a 6 degree head-down tilt from weeks to months in time^6^. There are reductions of bone mass in cancellous bone areas, with losses of 1.0% in the distal femur, 3.0% in the patella, and 2.0% in the distal tibia in a 35 day bed rest trial. After 56 days and 90 days, bone mass in the distal tibia declines 3.6% and 6.0% correspondingly^7^. For animal studies, the hind-limb suspension model is commonly used for rats and the effect of weightlessness on hind limbs is produced^8^. Hindlimb unloading decreases the number of osteoblasts in the metaphysis of the tibia after 5 days, and reduces the trabecular bone volume by 14 days^9, 10^. Experiments undertaken with clinostat devices using osteogenic cell cultures facilitate the evaluation of the biological effects of microgravity on the functional activities of osteoblasts^11, 12^.

A random positioning machine (RPM) is a particular kind of 3D clinostat with two independently rotating frames driven by independent motors, which has been successfully used to simulate microgravity^13^. One frame is positioned inside the other giving a very complex net change of orientation to a biological specimen. From the sample’s point of view, the gravity vector’s trajectory averaged over time shall converge toward zero. As a result, the biological specimen will experience a state similar to microgravity^14–16^. Researches have demonstrated that microgravity simulated by a RPM suppresses the expression levels of various osteogenesis makers, including alkaline phosphatase (ALP), runt-related transcription factor 2 (Runx-2), osteocalcin (OCN) and type 1 collagen (COL-1) and formation of mineralized nodules in cultured MC3T3 or 2T3 preosteoblasts and other osteoblastic cells^11, 17–19^. However, the mechanisms of microgravity-inhibited osteogenesis are almost completely unclear as this process is multistage and complex.

Primary cilium is a solitary organelle that emanates from the surface of most mammalian cells, which has been proved as a key coordinator of signaling pathways to respond mechanical and chemical stimuli. Since a variety of receptors, ion channels and transporter proteins have been localized to the cilium^20, 21^, its role as a mechanosensor has been established. Primary cilium will change its orientation in response to mechanical stimuli and trigger biochemical and transcriptional changes. This phenomenon has been identified and confirmed in kidney epithelial cells, osteocytes, mesenchymal stem cells, and cholangiocytes^22–25^. For example, mechanotransduction of fluid flow bends the primary cilium to induce specified gene expression by decreasing cAMP contents in osteocytes or activating calcium channel to mediate intracellular calcium mobilization in kidney cells^25, 26^. Defects in primary cilium structure or mutations disrupting ciliogenesis will lead to a variety of developmental abnormalities and postnatal disorders, including oral-facial-digital syndromes, Bardet-Biedl syndromes and ossification disorders^27–29^.

Recent studies have demonstrated that some pharmacological agents and extracellular environmental changes can alter the primary cilia length. Lithium treatment causes primary cilia to extend in several cell types via the inhibition of glycogen synthase kinase-3β (GSK3β) or adenylate cyclase III (ACIII)^30, 31^. Cytochalasin D, an inhibitor of actin polymerization, changes the microtubule network with a subsequent increase in soluble tubulin levels and results in a primary cilium elongation^32, 33^. Besides, it is widely recognized that there is a balance between cilia length and intraflagellar transport (IFT). The IFT particles and their associated cargo proteins are transported along axonemal microtubules by kinesin-2 motor proteins in the anterograde direction and by cytoplasmic dynein-2 in the retrograde direction. Blocking dynein-2 function by depleting dynein light chain 1 (DYNLT1) causes an increase in cilia length^34–36^.

Inspired by its established role as a mechanosensor, we hypothesize that the primary cilium is a critical target of microgravity stimuli. Along with the extension ability, the primary cilium may become a potential candidate against bone loss. To verify this hypothesis, we examined the changes in behavior of primary cilia of rat calvarial osteoblasts (ROBs) responding to RPM treatment, and analysed effects on osteogenesis by determining the differentiation, maturation and mineralization of osteoblasts. The effects of modulating formation or length of primary cilia on osteogenesis in a microgravity condition were also investigated using pharmacological agents or molecular approaches. Our results demonstrated that primary cilia played an important role in keeping the normal state of osteoblasts in microgravity condition.

## Results

### Simulated microgravity abrogates the primary cilia of ROBs

**Figure 1:**
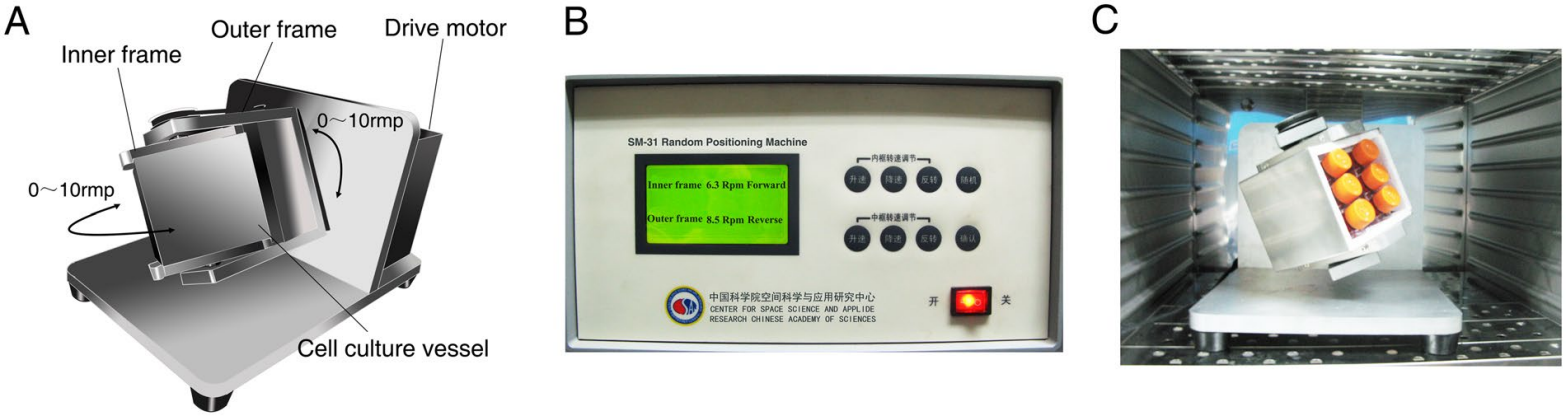
The random positioning machine (RPM) used in this study to generate microgravity for cultured cells. (**A**) The schematic view of the desktop RPM. (**B**) A photo of the control console operating the outer and inner frames of the RPM to rotate the cell culture vessel separately and randomly at 3-dimension in the range of 0–10 rpm. (**C**) A photo showing the RPM placed in a cell incubator. Six 50 ml-flasks with cultured ROBs sealed by aerated hydrophobic membrane were fixed on the cell culture vessel of the RPM.

**Figure 2:**
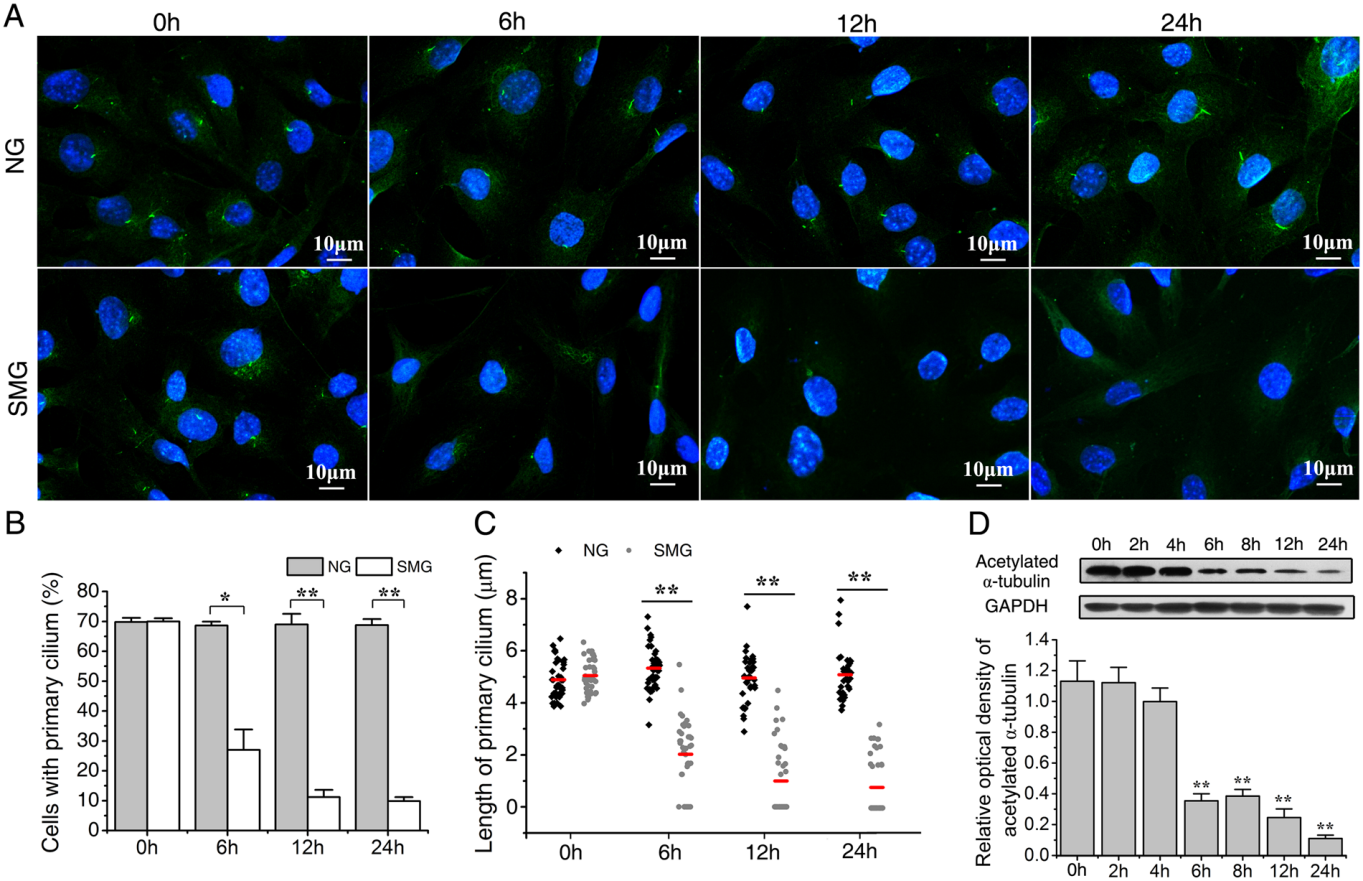
Simulated microgravity significantly abrogates the primary cilia of rat calvarial osteoblasts (ROBs). (**A**) The immunofluorescence image of ROBs with or without random positioning machine (RPM) microgravity treatment. Primary cilia were stained by acetylated α-tubulin (green), and the nuclei were stained with DAPI (blue). (**B**) The percentage of ciliolated cells with or without RPM treatment. (**C**) Means (red bars) and individual measurements of cilium lengths with or without RPM treatment. (**D**) The protein expression levels of acetylated α-tubulin in ROBs with or without RPM treatment. NG: normal ground, SMG: simulated microgravity. Full-length Western blots are presented in Supplementary Figure 1. Each experiment was conducted at least three times independently. *P < *0.05 or* **P < *0.01 vs* NG group or 0 h group.

Enlightened by the response of primary cilia to mechanical stimuli, we speculate that microgravity impacts the primary cilium. ROBs were treated with RPM (Fig. 1A–C) for 6 h, 12 h and 24 h respectively. The cilia were investigated under a laser confocal microscopy after immunofluorescence staining of acetylated α-tubulin, the main component of cilia. Surprisingly, it was found that the number of ROBs with primary cilia was greatly decreased after 6 h of RPM treatment, and continued to decline with time (Fig. 2A). The residual primary cilia became obviously shorter and dotted. Compared to the normal ground group, simulated microgravity caused a reduction of 60.5 ± 10.4% at 6 h (P < 0.05), 83.7 ± 4.4% at 12 h (P < 0.01) and 85.7 ± 1.9% at 24 h (P < 0.01) in the percentage of cells with primary cilia (Fig. 2B). The average length of cilia changed from 5.11 ± 0.89 μm (normal ground group, NG) to 0.79 ± 0.18 μm (simulated microgravity group, SMG) at 24 h (P < 0.01, Fig. 2C).

To further verify the disrupting effect of microgravity on primary cilia, the total protein levels of acetylated α-tubulin were examined at 0 h, 2 h, 4 h, 6 h, 8 h, 12 h and 24 h after RPM treatment. As shown in Fig. 2D, although there were no significant differences between 0 h group and SMG groups at 2 h and 4 h, the acetylated α-tubulin levels were significantly reduced at 6 h, and continued to decrease up to 24 h (P < 0.01), implying a general suppression effect on the acetylated α-tubulin of osteoblasts in the microgravity condition. All the above results demonstrate that microgravity exerts a detrimental effect on the primary cilia of ROBs.

### Osteogenesis of ROBs is reduced by simulated microgravity

**Figure 3:**
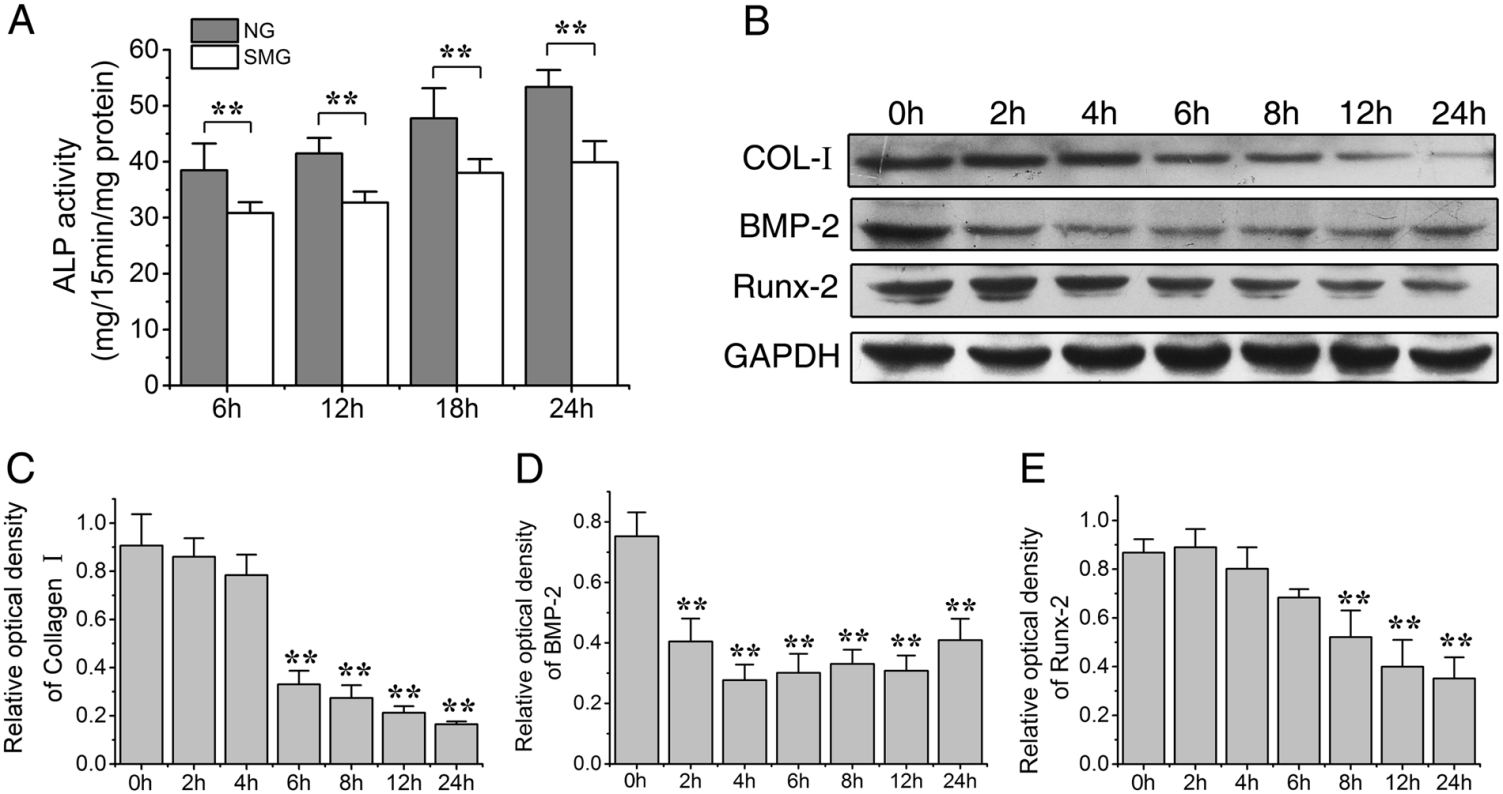
Simulated microgravity inhibits the osteogenic differentiation of rat calvarial osteoblasts (ROBs). (**A**) The alkaline phosphatase (ALP) activities after ROBs were exposed to simulated microgravity (SMG) and normal ground (NG) for different time periods. (**B**) Representative Western blots detecting the expression levels of osteogenesis markers, COL-I, BMP-2 and Runx-2 (with GAPDH as a control) in ROBs at different time points after random positioning machine (RPM) exposure. (**C**) Relative protein levels of COL-I, BMP-2 and Runx-2. NG: normal ground, SMG: simulated microgravity. Full-length Western blots are presented in Supplementary Figure 2. Each experiment was conducted at least three times independently. *P < *0.05 or* **P < *0.01 vs* NG group or 0 h group.

To determine whether simulated microgravity affects osteogenic differentiation of ROBs, the ALP activities were measured after ROBs were exposed to 6 h, 12 h and 24 h of RPM treatment. As shown in Fig. 3A, the ALP activities of SMG groups were significantly lower than those of NG groups (*P* < 0.01), and kept at lower levels up to 24 h. Protein levels of osteogenic makers including Runx-2, bone morphogenetic protein-2 (BMP-2), and COL-I were examined at 0 h, 2 h, 4 h, 6 h, 8 h, 12 h and 24 h after RPM treatment, respectively. It was found that protein levels of COL-I in SMG groups at 6 h, 8 h, 12 h and 24 h became significantly lower than that of 0 h group control (*P* < 0.01, Fig. 3B,C). BMP-2 levels in SMG groups became significantly lower than 0 h group at 2 h (P < 0.01, Fig. 3B,D), and kept at an extremely low level at other time points. Levels of Runx-2 declined with increasing time in RPM treatment, which became significantly lower than 0 h group control after 8 h, 12 h and 24 h (P < 0.01) (Fig. 3B,E).

**Figure 4:**
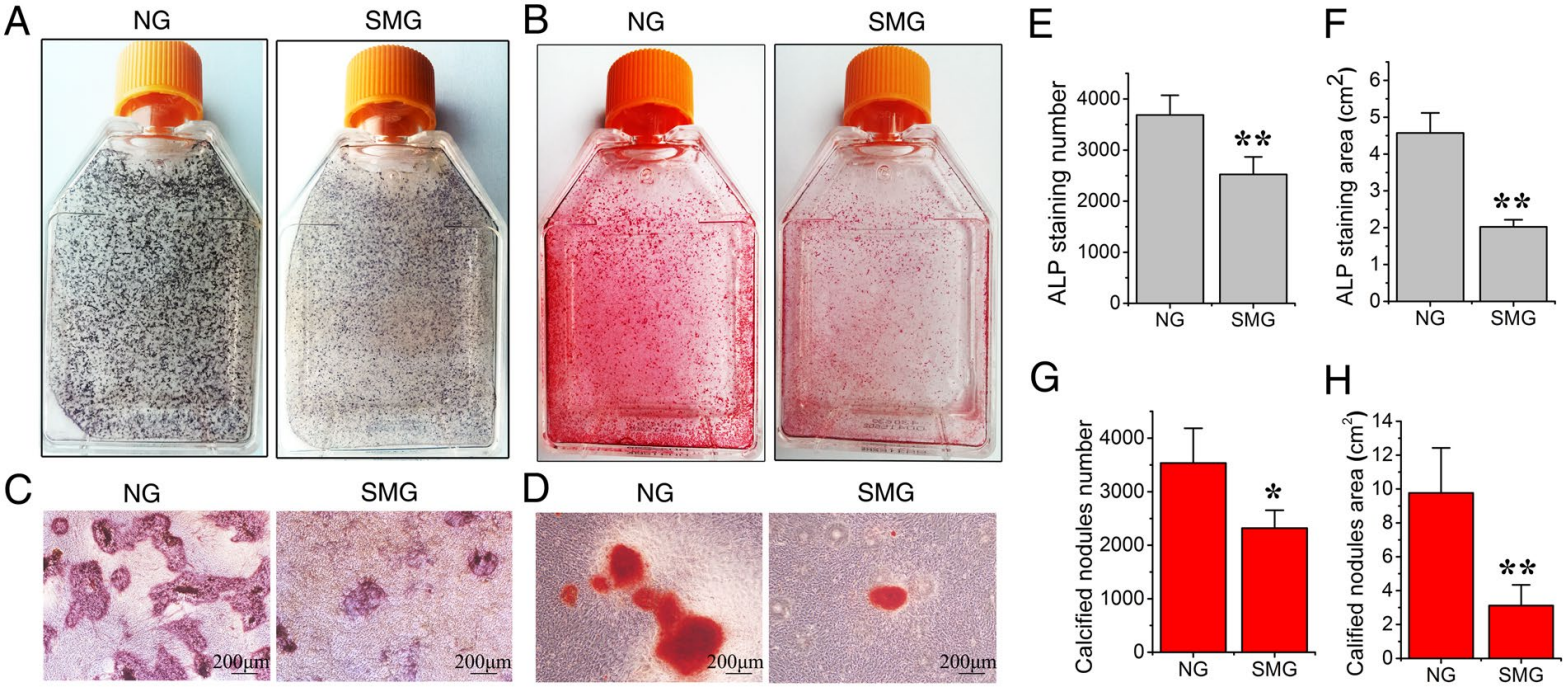
Simulated microgravity reduces the maturation and mineralization of rat calvarial osteoblasts (ROBs). (**A**) Representative image of the alkaline phosphatase (ALP) stained CFU-F_ALP_ colonies after 8 days of random positioning machine (RPM) microgravity treatment. (**B**) Representative image of the stained mineralized nodules after 12 days of RPM treatment. (**C**) Enlarged image of the CFU-F_ALP_ colonies under a phase contrast microscope. (**D**) Enlarged image of the mineralized nodules under a phase contrast microscope. (**E** and **F**) The numbers and areas of the CFU-F_ALP_ . (**G** and **H**) The numbers and areas of the mineralized nodules. NG: normal ground, SMG: simulated microgravity. Each experiment was conducted at least three times independently. *P < *0.05 or* **P < *0.01 vs* NG group.

To examine whether simulated microgravity suppressed the maturation and mineralization of ROBs, cultured cells in osteogenic induction medium were exposed to 6 h of RPM treatment per day. The ALP-positively stained colonies (CFU-F_ALP_) and alizarin red-stained mineralized nodules were analyzed after 8 days and 12 days of RPM treatment, respectively. The results of ALP histochemical staining showed that the number and area of CFU-F_ALP_ colonies in SMG group were significantly lower than those in the NG group (P < 0.01, Fig. 4A,C,E and F). Alizarin red staining results demonstrated that RPM treatment significantly decreased the number and area of mineralized nodules compared with the NG group (P < 0.05, Fig. 4B,D,G and H). Based on these results, it can be concluded that the simulated microgravity inhibits the osteogenic differentiation, maturation and mineralization of ROBs.

### Cytochalasin D prevents the primary cilium abrogation induced by simulated microgravity

As we observed that primary cilia of ROBs gradually shrank and almost completely disappeared after RPM exposure, we further investigated if microgravity-induced abrogation of primary cilia and reduction of osteogenesis could be prevented by appropriate pharmacological agents.

**Figure 5:**
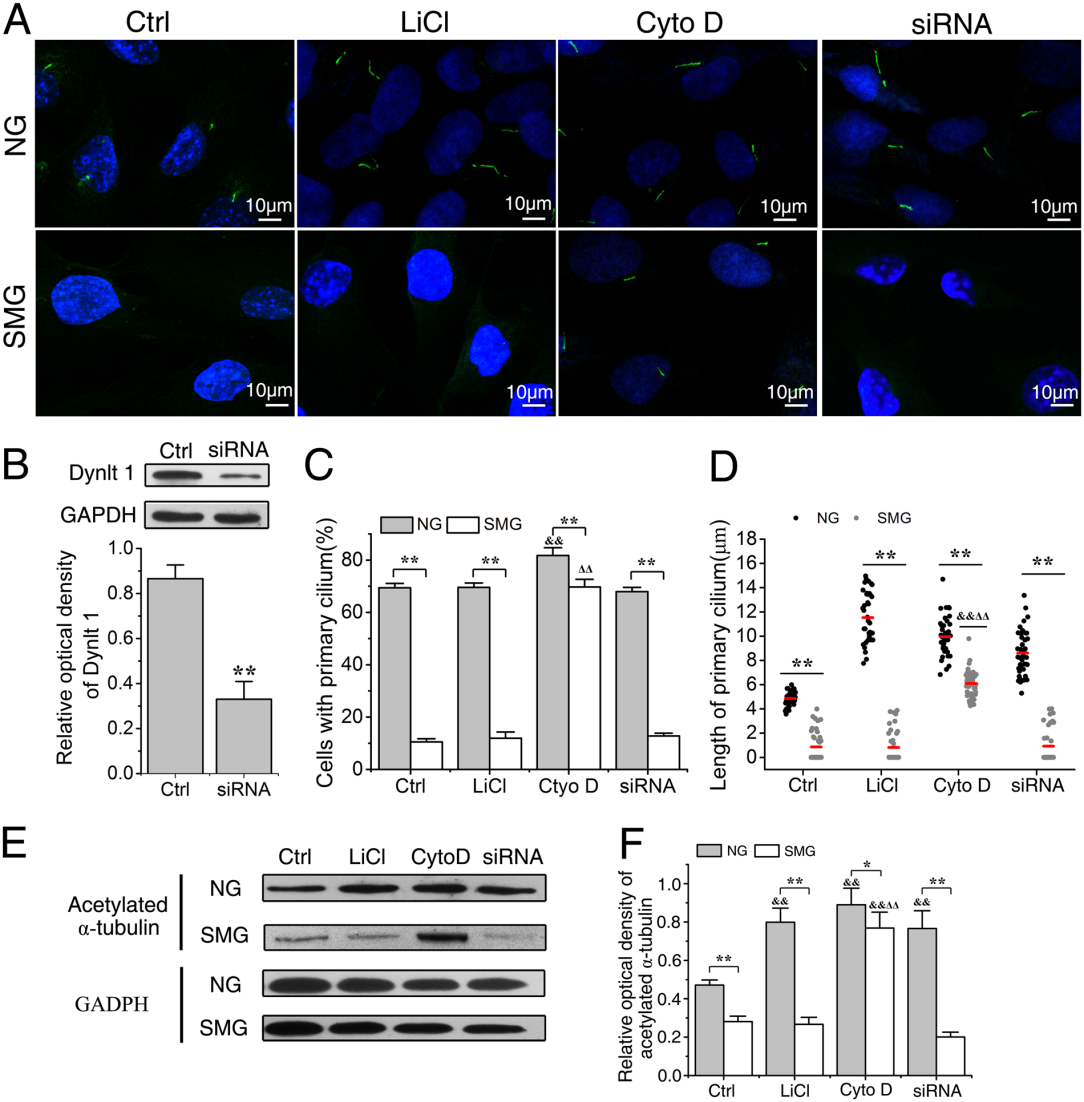
Elongation of cilia prevents the abrogation of primary cilia induced by simulated microgravity. (**A**) Immunofluorescence image of rat calvarial osteoblasts (ROBs) exposed to simulated microgravity (SMG) or normal ground (NG) for 24 h after being treated with 5 mM LiCl, 0.1 μM cytochalasin D (Cyto D) or dynein light chain Tctex-type 1 (Dynlt1) siRNA. Primary cilia were stained with acetylated α-tubulin (green), and DNA were stained with DAPI (blue). (**B**) Treatment with Dynlt1 siRNA resulted in significant decreases in Dynlt1 expression at the protein levels. (**C**) Quantification of the percentage of cells with a cilium in each group. (**D**) Means (red bars) and individual measurements of cilium lengths in each group. (**E**) The protein expression levels of acetylated α-tubulin. Full-length Western blots are presented in Supplementary Figure 3 and Figure 4. Each experiment was conducted at least three times independently. *P < *0.05 or* **P < *0.01 vs* NG group of each treatment; ^*&*^ *P* < 0.05 or ^*&&*^ *P* < 0.01 vs Ctrl of NG group; ^△△^ *P* < 0.01 vs Ctrl of SMG groups.

First, we checked the lengths of primary cilia in the normal ground condition after ROBs were treated with 5 mM LiCl, 0.1 μM cytochalasin D (Cyto D, an inhibitor of actin polymerization), or siRNA of Dynlt1 (dynein light chain Tctex-type 1), which were all reported to be able to elongate the primary cilia by different mechanisms^30, 32–34, 36^. As shown in Fig. 5, all treatments increased the lengths of primary cilia to some extents (Fig. 5A,C,D). Lithium elongated primary cilia from 4.86 ± 0.58 μm (Ctrl group) to 11.62 ± 1.29 μm (LiCl group). Particularly, some cilia even reached a maximal length of 14.52 μm. The lengths of primary cilia in Cyto D group reached an average of 9.96 ± 2.29 μm (P < 0.01, compared with Ctrl). Dynlt1 siRNA showed the weakest elongating ability but still significantly increased the lengths of primary cilia to 8.62 ± 1.83 μm (P < 0.01, compared with Ctrl). Besides, Cyto D significantly increased the fraction of cells with primary cilia from 69.4 ± 1.7% to 81.7 ± 3.0%, while no such effects were found in LiCl and Dynlt1 siRNA groups.

Next, we examined the effects of ciliogenesis intervention treatments on the lengths of primary cilia in the microgravity condition. The primary cilia in Ctrl, LiCl and Dynlt1 siRNA groups became obviously shorter and dotted (compared with NG group of each treatment, P < 0.01) (Fig. 5A,C,D), with a reduction of 83.7 ± 3.2% in the percentage of cells displaying primary cilia (from 69.6 ± 1.6% to 11.9 ± 2.4%, P < 0.01) in the LiCl group and 82.0 ± 1.8% reduction (from 67.9 ± 1.6% to 12.8 ± 1.1%, P < 0.01) in the Dynlt1 siRNA group. However, the primary cilia were maintained in the Cyto D group, with only a reduction of 14.9 ± 0.9% in the percentage of cells displaying primary cilia (from 81.7 ± 3.0% to 69.7 ± 2.9%). In addition, the cells in the Cyto D group displayed significantly longer primary cilia (with an average length of 6.10 ± 1.13 μm) than those in the Ctrl of SMG group (0.84 ± 1.24 μm, P < 0.01), which were also longer than cilia of cells in Ctrl of NG group (4.86 ± 0.58 μm, P < 0.01).

Then, we further analysed the treatment effects on primary cilia by examining the total protein levels of acetylated α-tubulin after LiCl, Cyto D or Dynlt1 siRNA treatment with or without RPM exposure. As shown in Fig. 5E and F, LiCl, Cyto D or Dynlt1 siRNA treatment significantly increased the acetylated α-tubulin levels in the normal ground condition (P < 0.01, compared with Ctrl of NG groups). However, the acetylated α-tubulin levels of Ctrl, LiCl and Dynlt1 siRNA groups were significantly decreased in the microgravity condition (P < 0.01, compared with NG group of each treatment). Strikingly, Cyto D treatment resulted in the highest acetylated α-tubulin level in SMG groups (P < 0.01, compared with Ctrl of SMG groups). Taken together, the above results suggest that Cyto D has the ability to prevent the abrogation of primary cilia induced by simulated microgravity.

### Cytochalasin D treatment offsets the reduction in osteogenesis caused by simulated microgravity

**Figure 6:**
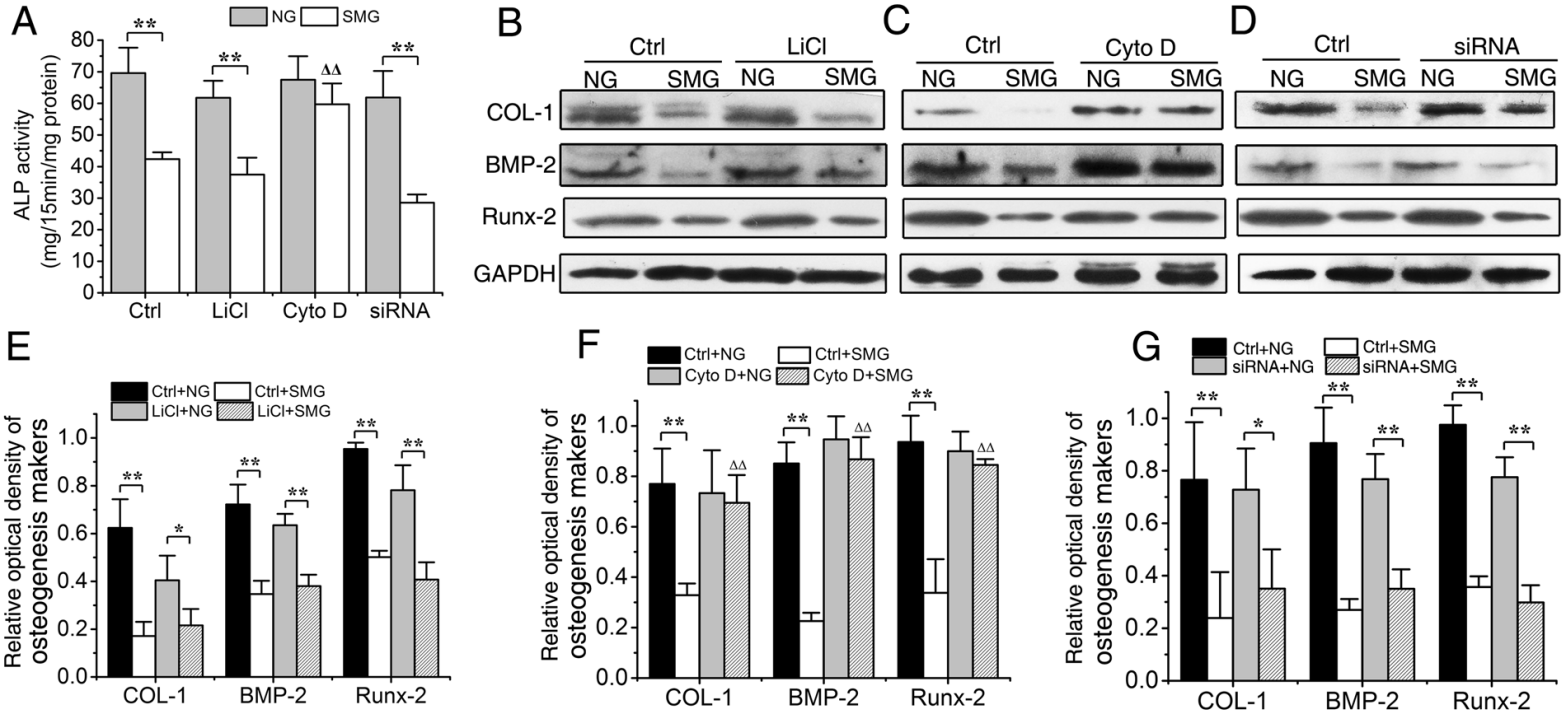
Elongation of cilia promotes the osteoblastic differentiation of rat calvarial osteoblasts (ROBs) in microgravity condition. (**A**) Alkaline phosphatase (ALP) activities of ROBs in Ctrl group, LiCl group, cytochalasin D (Cyto D) group or dynein light chain Tctex-type 1 (Dynlt1) siRNA group with or without random positioning machine (RPM) microgravity exposure. (**B**) Representative Western blot detecting expression of osteogenesis markers COL-I, BMP-2 and Runx-2 (with GAPDH as a control) in each group. (**C**) The protein levels of COL-I, BMP-2 and Runx-2 in each group. NG: normal ground, SMG: simulated microgravity. Full-length Western blots are presented in Supplementary Figure 5. Each experiment was conducted at least three times independently. *P < *0.05 or* **P < *0.01 vs* NG group of each treatment; ^△△^ *P* < 0.01 vs Ctrl of SMG groups.

After observing the impairment of osteogenesis following RPM exposure and the possibility of preventing the SMG-induced primary cilium abrogation, we investigated whether microgravity- reduced osteogenesis could be prevented by the preservation of primary cilia. As shown in Fig. 6A, the ALP activities of Ctrl, LiCl and Dynlt1 siRNA groups were reduced dramatically by RPM treatment (compared with NG groups, P < 0.01), whereas no significant differences were found between SMG group and NG group treated with Cyto D (P = 0.14). Consistently, while the protein levels of COL-I, BMP-2 and Runx-2 in Ctrl, LiCl and Dynlt1 siRNA groups were all greatly decreased in the microgravity condition when compared with those in normal ground condition (P < 0.01), no such significant reductions were detected in Cyto D treatment group (COL-I, P = 0.72; BMP-2, P = 0.25 and Runx-2, P = 0.50, Fig. 6B–G).

**Figure 7:**
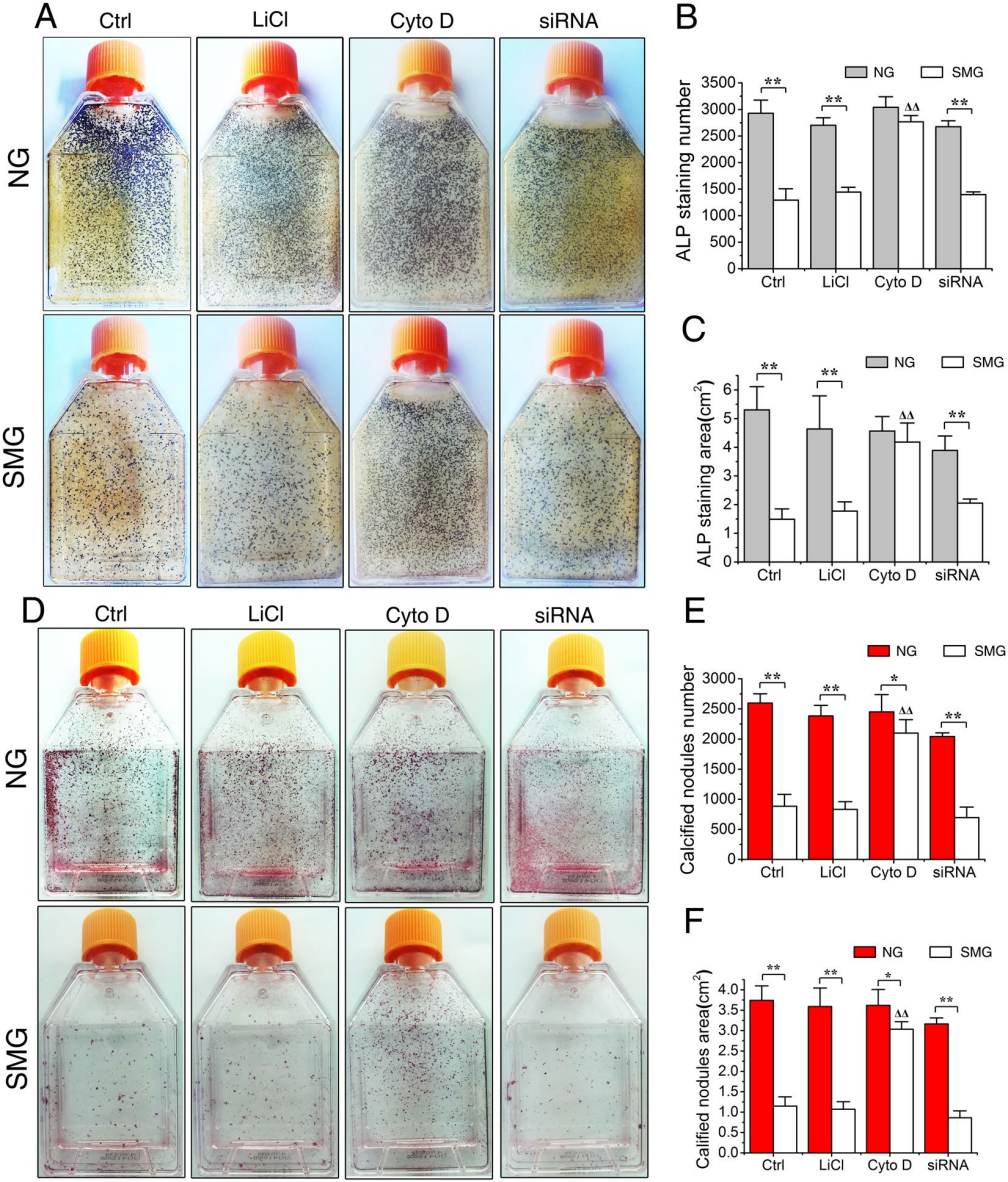
Elongation of cilia promotes the maturation and mineralization of rat calvarial osteoblasts (ROBs) in microgravity condition. (**A**) Representative images of alkaline phosphatase (ALP) stained CFU-F_ALP_ colonies after 8 days in Ctrl group, LiCl group, cytochalasin D (Cyto D) group or dynein light chain Tctex-type 1 (Dynlt1) siRNA group with or without random positioning machine (RPM) microgravity exposure. (**B** and **C**) The numbers and areas of CFU-F_ALP_ in each group. (**D**) Representative image of stained mineralized nodules after 12 days in each group. (**E** and **F**) The numbers and areas of mineralized nodules in each group. NG: normal ground, SMG: simulated microgravity. Each experiment was conducted at least three times independently. *P < *0.05 or* **P < *0.01 vs* NG group of each treatment; ^△△^ *P* < 0.01 vs Ctrl of SMG groups.

The results of ALP histochemical staining after 8-day of simulated microgravity exposure confirmed that, whilst simulated microgravity evidently reduced the CFU-F_ALP_ colony numbers and areas in Ctrl, LiCl and Dynlt1 siRNA groups (Fig. 7A–C, P < 0.01), Cyto D treatment offset the inhibition in CFU-F_ALP_ colony formation as no significant decreases in the number (P = 0.05) and area (P = 0.47) were found between SMG group and NG group. Similarly, results of mineralized nodule staining after 12-day osteogenic culture showed that, while simulated microgravity significantly decreased the numbers and areas of mineralized nodules in Ctrl, LiCl and Dynlt1 siRNA groups (Fig. 7D–F, P < 0.01), the number and area of mineralized nodules in Cyto D group treated by RPM were significantly higher than those in Ctrl (P < 0.01) and other groups under the microgravity condition. The above results suggest that simulated microgravity-induced inhibition on osteogenesis can be rescued by Cyto D treatment.

### IFT88 siRNA treatment inhibits the ability of cytochalasin D to counteract the reduction of osteogenesis

**Figure 8:**
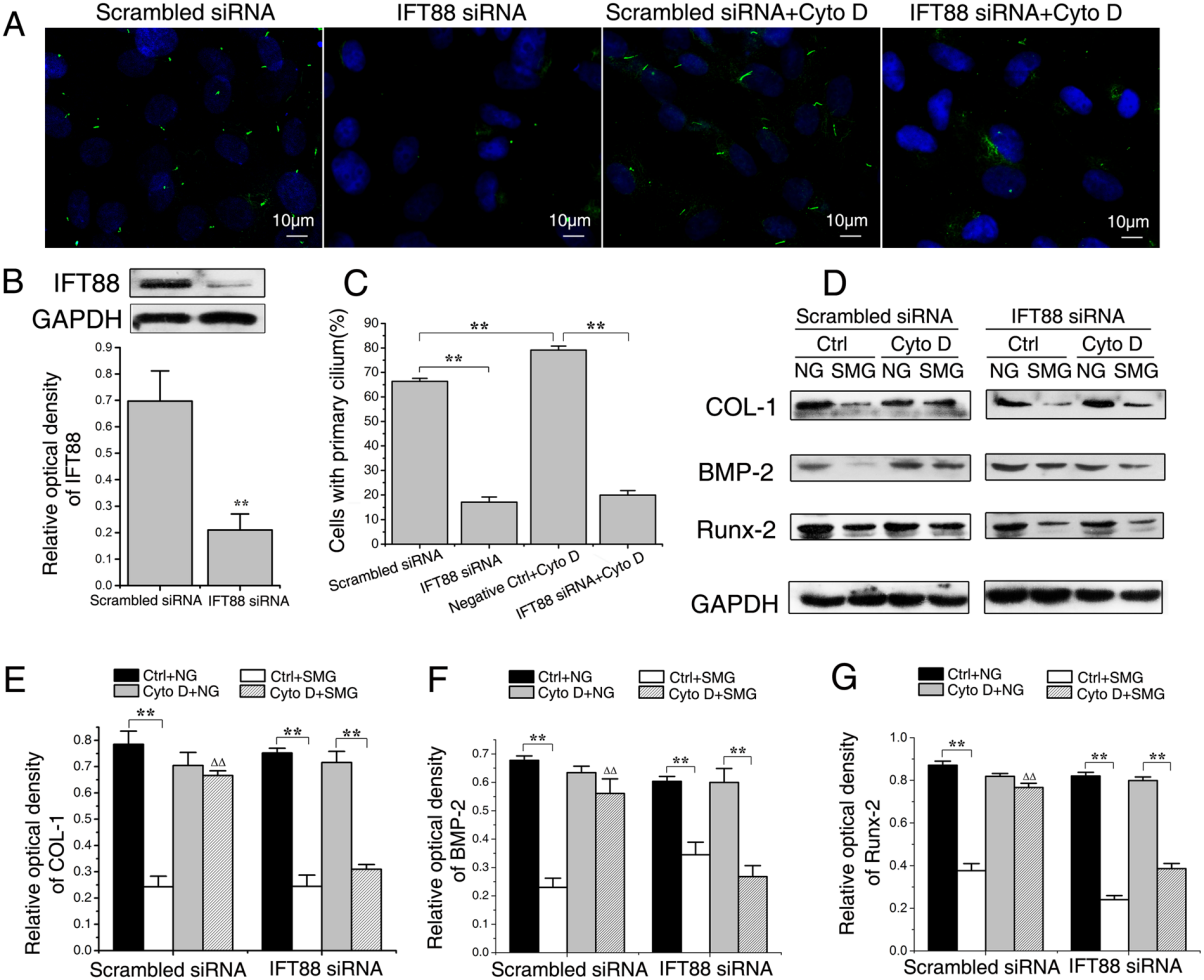
Intraflagellar transport protein 88 (IFT88) siRNA knockdown inhibits the ability of cytochalasin D (Cyto D) to counteract the reduction of osteogenesis. (**A**) Immunofluorescence image of rat calvarial osteoblasts (ROBs) treated with or without IFT88 siRNA and 0.1 μM Cyto D. Primary cilia were stained with acetylated α-tubulin (green), and DNA were stained with DAPI (blue). (**B**) Treatment with IFT88 siRNA resulted in significant decreases in IFT88 expression at the protein levels. (**C**) Quantification of the percentage of cells with a cilium in each group. (**D**) Representative Western blot detecting expression of osteogenesis markers COL-I, BMP-2 and Runx-2 (with GAPDH as a control) in each group. (**E**-**G**) The protein levels of COL-I, BMP-2 and Runx-2 in each group. NG: normal ground, SMG: simulated microgravity. Full-length Western blots are presented in Supplementary Figures 6 and 7. Each experiment was conducted at least three times independently. **P < *0.01 vs* NG group of each treatment; ^△△^ *P* < 0.01 vs Ctrl of SMG groups.

To confirm the ability of Cyto D in offsetting microgravity-induced osteogenesis repression is due to the preservation of primary cilia, we blocked ciliogenesis using siRNA sequence targeting IFT88, an essential component for the assembly and maintenance of primary cilia, and then examined treatment effects on the differentiation of ROBs in simulated microgravity condition with Cyto D. IFT88 siRNA treatment for 24 h significantly decreased the protein expression levels of IFT88 (P < 0.01 compared to scrambled siRNA control). As expected and as examined by immunofluorescence staining, after IFT88 siRNA treatment, primary cilia became dotted and obviously shorter (Fig. 8A and B), and the number of ROBs with primary cilia was reduced from 66.4 ± 1.2% to 17.0 ± 2.1% (P < 0.01, Fig. 8C). After the treatment of Cyto D, primary cilia of scrambled siRNA group (scrambled siRNA + Cyto D) became significantly longer than the scrambled siRNA Ctrl group (scrambled siRNA+Ctrl), and Cyto D treatment significantly increased the fraction of cells with primary cilia from 66.4 ± 1.2% to 79.1 ± 1.7% (P < 0.01, Fig. 8A and C). However, after the IFT88 siRNA treatment, the elongation ability of Cyto D treatment on primary cilia was inhibited (Fig. 8A), and the fraction of cells with primary cilia was decreased from 79.1 ± 1.7% (scrambled siRNA Ctrl+Cyto D group) to 19.9 ± 1.9% (IFT88 siRNA+Cyto D group) (P < 0.01, Fig. 8A and C).

As shown in Fig. 8D–G, for the scrambled siRNA Ctrl group, the protein levels of COL-I, BMP-2 and Runx-2 were all greatly decreased in microgravity condition when compared with those in normal ground condition (P < 0.01). After the treatment with Cyto D (which preserves primary cilia), no such significant reductions were detected (COL-I, P = 0.90; BMP-2, P = 0.28 and Runx-2, P = 0.11, Fig. 8D–G). However, for the IFT88 siRNA group, no matter with or without Cyto D treatment, the protein levels of COL-I, BMP-2 and Runx-2 were all decreased in the microgravity condition when compared with those in the normal ground condition (P < 0.01, Fig. 8D–G). The above results suggest that while Cyto D treatment could maintain primary cilia and effectively offset the reduction in osteogenesis caused by microgravity, suppression of such effects by inhibiting ciliogenesis using IFT88 siRNA confirms the central role of primary cilium preservation in the ability of Cyto D to maintain osteogenesis under the microgravity condition. Thus, primary cilia could be a potential target against the reduction of osteogenesis induced by simulated microgravity.

## Discussion

Astronauts suffer from distinct loss of bone mineral density (BMD) during the spaceflight, which has been shown to closely mimic osteoporosis^37^. In missions aboard the international space station, seven of eight astronauts experienced a decrease in BMD in the range of 2.5–10.6% in the lumbar vertebrae. All eight astronauts experienced a loss of total BMD in the range of 3.0–10.0% in the femur and four of the eight had 1.7–10.0% loss in the femoral neck^38, 39^. Spaceflights led to remarkable reductions of periosteal bone formation and longitudinal bone growth in growing rats, and decreases in osteoblast number and activity which were likely the result of altered differentiation of osteoblast precursors^40, 41^. However, the underlying mechanisms of microgravity-induced osteopenia are still unclear. Without a total understanding of the pathophysiology, effective prevention or treatment of microgravity-induced bone loss is unlikely.

Bone alters its morphology and density in response to external loads. A lack of mechanical stimulation has been linked to bone loss in osteoporosis^42^. However, the mechanism for this link is unclear. The primary cilium has been proposed to function as a mechanosensor to translate loading-induced signals into biochemical and transcriptional responses. Previous studies have demonstrated that primary cilia play an indispensable role in osteogenesis process^43^. Yan *et al*. found that blocking ciliogenesis using siRNA-mediated depletion of IFT88 inhibited the promoting effect of pulsed electromagnetic fields (PEMFs) on osteogenic differentiation of ROBs^44^. Delaine *et al*. reported that oscillatory fluid flow (OFF) significantly reduced the number of osteoblasts expressing primary cilia, and that damage or removal of primary cilia using chloral hydrate inhibited OFF-induced PGE_2_ release and mineral deposition^45^. In the present work we identified, for the first time, that primary cilia of ROBs were almost completely depleted after 24 h of RPM microgravity treatment. Microgravity-induced inhibition of osteogenic differentiation, maturation and mineralization of ROBs were closely associated with the abrogation of primary cilia. These results indicate that RPM-induced disrupting of primary cilia may play a key role in microgravity-induced reduction in osteogenesis.

It has been found that microgravity affects the cytoskeleton in various cells. Gershovich *et al*. reported that actin cytoskeleton of mesenchymal stem cells (MSCs) was found to be reorganized rapidly even after 30 minutes of simulated microgravity, and that the number of cells with disrupted actin cytoskeleton steadily increased with the exposure time^46^. Cyto D was widely known for its disrupting effects on actin cytoskeleton, and was found to be a modulator of ciliogenesis and cilium length, firstly by Kim *et al*. and confirmed later by many others^33, 47^. Sharma *et al*. reported that Cyto D increased the cilia length through changes in the actin network and in levels of soluble tubulin^32^. Sen *et al*. found that depolymerization of actin cytoskeleton in MSCs using Cyto D induced a robust osteogenic gene expression program in bone marrow-derived MSCs and intra-tibial injection of CytoD induced abundant trabecular and even cortical bone formation after only one week of injection^48^.

Studies have found that microgravity-induced changes in actin cytoskeleton are responsible for the reduction in osteogenesis. In particular, Dai *et al*. reported that actin microfilaments participate in BMP-2 activity in activating Runx-2 and that their disruption might be an important contributor to microgravity-induced inhibition on BMP-2 activity in osteogenic induction^49^. In the present study, we found that microgravity-induced abrogation of primary cilia may be another underlying mechanism for the microgravity inhibitory effect on osteogenesis. We have shown that Cyto D effectively maintained the existence of primary cilia in the microgravity condition and offset the reduction in osteogenesis caused by RPM exposure, and that blocking ciliogenesis using siRNA knockdown of IFT88 inhibited the ability of cytochalasin D to counteract the reduction of osteogenesis. However, it has been known that there are intrinsic connections between cytoskeleton changes and primary cilia. For example, Espinha *et al*. found that primary cilia could regulate alterations in the cytoplasmic microtubule network in response to mechanical stimulation^50^. Therefore, future work is required to determine whether microgravity affects actin cytoskeleton through primary cilia or abrogates the primary cilia by changing the actin network.

In summary, microgravity-induced inhibition of osteogenic differentiation, maturation and mineralization was closely associated with the abrogation of primary cilia. Cytochalasin D treatment could maintain primary cilia and effectively offset the reduction in osteogenesis caused by simulated microgravity. Primary cilia could be a potential therapeutic target against bone loss during space flights, and perhaps even disease-related bone loss due to immobilization and physical inactivity.

## Materials and Methods

### Isolation and culture of rat calvarial osteoblasts (ROBs)

Primary osteoblasts (ROBs) were isolated from the skull bones as described^44^ of neonatal SD rats obtained from the Laboratory Animal Center, Gansu University of Chinese Medicine (Lanzhou, China). Animal experiments were approved by Animal Ethics Committee of Lanzhou General Hospital and were conducted according to NIH Guidelines for the Care and Use of Laboratory Animals. In brief, calvarias were dissected aseptically, cleaned of adhering soft tissues, minced to small pieces, digested at 37 °C with 0.25% trypsin twice, 15 min for each time, and then with 1 mg/mL collagenase II (Sigma, St. Louis, MO, USA) six times, 20 min for each time. The released cells from the last four digest supernatants were pooled and filtered through a 200-μm sieve to remove bone debris. Cells were collected by centrifugation at 1000 rpm for 5 min, and resuspended in α-MEM (Gibco, Gaithersburg, MD, USA) plus 10% fetal calf serum (Gibco, Gaithersburg, MD, USA). The cells were seeded in flasks sealed with an aerated hydrophobic membrane. Flasks, incubated in a humidified atmosphere of 5% CO_2_ at 37 °C, were completely filled with fresh culture medium in order to prevent air bubbles and to minimize liquid flow. The cells from the first to third passages were used in the following experiments.

### Random position machine

The SM-31 random positioning machine (RPM) (Center for Space and Applied Research, Chinese Academy of Science, Lanzhou, China) which was used to simulate microgravity consists of a desktop RPM, a control console and a temperature sensors (Fig. 1). The RPM contains two independent rotating frames. One frame is positioned inside the other giving a very complex net change of orientation to a biological sample mounted in the middle. Both frames can rotate randomly at 3-dimension in the range of 0–10 rpm with changes in the acceleration and direction of the biological sample over time. The RPM was placed in a CO_2_ incubator (5% CO_2_, 37 °C and 100% humidity) to exposure biological samples to microgravity and it was connected to the control console outside of the incubator. In this study, ROBs were cultured in 50-ml flasks with an aerated hydrophobic membrane. After 24 h, flasks were completely filled with fresh culture medium and fixed on a 12.3 (length) × 9.8 (width) × 10 cm (height) cell culture vessel for RPM exposure. ROBs in the normal ground (NG) group were also seeded in flasks filled with fresh culture medium and were statically placed in the same CO_2_ incubator with the RPM.

### Alkaline phosphatase (ALP) activity measurement

After different time periods of RPM exposure, cells were lysed and homogenized in RIPA lysis buffer (Applygen, Beijing, China). Supernatants were obtained by centrifugation at 4 °C for 30 min at 12,000 rpm, and the protein contents were quantified by a BCA kit (Thermo Fisher Scientific, Waltham, MA). The ALP activities were measured biochemically with a modified method of King using a commercial kit as instructed (Nanjing Jiancheng Bioengineering Ltd, Nanjing, China). Results of ALP activities were expressed as nmol phenol/15 min/mg protein.

### ALP histochemical staining and mineralized nodular staining

The numbers of colonies formed and positive for ALP (CFU-F_ALP_) were evaluated after the osteoblasts had been exposed to microgravity for 8 days at 6 h/day. The flasks with osteoblasts were washed and fixed in 4% paraformaldehyde and then stained for 15–20 min at 37 °C with 20 ml pH 8.9 Michalis buffer, containing 10 mg 1-naphthyl phosphate sodium and 20 mg fast blue RR salt. Calcified nodules in flasks were assessed after 12 days of microgravity treatment at 6 h/day. Cell cultures were fixed with 4% paraformaldehyde and stained for 30 min with Alizarin red at 37 °C. The number and area of CFU-F_ALP_ or Alizarin red-stained mineralized nodules were measured with Image-Pro Plus 6.0.

### Immunofluorescence microscopy and length measurements of primary cilia

Cells were fixed in 4% paraformaldehyde and permeabilised in PBS with 0.1% Triton X-100 for 10 min. After a brief wash, they were incubated for 1 h in 1% bovine serum albumin. Immunostaining was carried out using primary antibodies targeted against acetylated α-tubulin (1:500, Abcam, Cambridge, UK) and the FITC-labeled goat anti-mouse IgG secondary antibody (1:500, KPL, Gaithersburg, MD). Nuclei were counterstained with DAPI (1:100, Sigma, St. Louis, MO). Cells were imaged under a laser-scanning confocal microscope (Olympus FV1000, Tokyo, Japan). The lengths of primary cilia were measured using Image-Pro Plus 6.0 software, by tracing the midline of primary cilia, with parameters (the ratio of fluorescent image pixel dimension *vs* actual size of an object in micrometer) predetermined using a stage objective micrometer. The number of ciliated cells and total number of cells were counted in a given field to determine the percentage of ciliated cells.

### Western blotting

Total cellular proteins (20 μg from each sample) obtained above were separated by SDS-PAGE (Solarbio, Beijing, China) under reducing conditions and transferred onto a nitrocellulose membrane (Invitrogen, Shanghai, China). Membranes were blocked in 5% dry milk and probed overnight at 4 °C with gentle rocking with anti-Runx-2 (1:1000, Abcam, Cambridge, UK), rabbit anti-BMP-2 (1:1000, Abcam), rabbit anti-COL-1 (1:1000, Abcam), mouse anti-acetylated α-tubulin (1:1000, Abcam), rabbit anti-Dynlt1 (1:800, Abcam), rabbit anti-IFT88 (1:500, Santa Cruz Biotech, Dallas, Texas), or rabbit anti-GAPDH (1:800, Bioworld, Shanghai, China). After washes, membranes were incubated with a secondary antibody (HRP-conjugated goat anti-rabbit IgG or goat anti-mouse IgG, 1:10000, Bioworld). The proteins recognized by the antibody complexes were visualized by chemiluminescence (Solarbio, Beijing, China). The optical density of each maker band was measured using Image-Pro Plus 6.0 software.

### Drug treatment experiments

To elongate the primary cilia, ROBs were treated with 5 mM lithium chloride (Sigma) or 0.1 μM Cyto D (Sigma) during the culture in the simulated microgravity condition or normal ground condition as described^30–33^. To evaluate the effect of primary cilia elongation on the osteogenic differentiation of ROBs, the cells were treated with or without drugs in osteogenic induction medium (growth medium supplemented with 10^−8 ^mM dexamethasone, 10 mM β-glycerol phosphate, 50 mg/ml ascorbic acid), and after treatment for different days, formation of the CFU-F_ALP_ colonies and mineralized nodules were analyzed as described above.

### RNA interference of Dynlt1 and IFT88

Dynlt1 is a canonical dynein motor responsible for the core functions of dynein dynein-2 which functions as the trafficking particles of primary cilia in the retrograde direction. The siRNA-mediated silencing of *Dynlt1* has been one of the methods to increase length of cilia^34, 36^. Briefly, siRNA targeting Dynlt1 (sequence: 5′-GAAGAATGGTGCTGGGTTA-3′) and a scrambled control siRNA were synthesized, cloned into U6 vectors (Invitrogen) and transfected into ROBs using lipofectamine 2000 (Invitrogen). Green fluorescent protein (GFP) was used as an indicator for success of transfection. Assays for evaluating Dynlt1 silencing efficiency were performed 24 h after transfection by Western blotting. To prevent cilium formation, ROBs were treated with a siRNA to knockdown the expression of intraflagellar transport 88 homolog (IFT88), a protein required for formation and assembly of cilia^20, 44^. For IFT88 silencing, one siRNA targeting IFT88 (sequence: 5′-GGAUAUGGGUCCAAG ACAUCC-3′) and a corresponding scrambled negative control siRNA (Invitrogen) were used. Assays for checking IFT88 silencing efficiency were carried out similarly as for Dynlt1 interference.

### Statistical analyses

Statistical analyses were performed using SPSS 20.0 (Chicago, IL, USA). Data are reported as means ± standard deviation. Cilia length measurements were conducted with n = 40 per each experimental group and other measurements were carried out with n = 3 for each treatment, and each experiment was repeated at least 3 times. Statistical differences between two groups of data were analyzed using Bonferroni modification of Student’ s *t*-test and ANOVA followed by a Dunnett’s or LSD post hoc test. *P* values less than 0.05 are considered to be significantly different.

## Electronic supplementary material

Supplementary information

## References

[1] Loomer PM (2001). The impact of microgravity on bone metabolism *in vitro* and *in vivo*. Crit. Rev. Oral. Biol. Med..

[2] Kramer LA, Sargsyan AE, Hasan KM, Polk JD, Hamilton DR (2012). Orbital and intracranial effects of microgravity: findings at 3-T MR imaging. Radiology..

[3] Leach CS (1992). Biochemical and hematologic changes after short-term space flight. Microgravity Q..

[4] Mccarthy ID (2005). Fluid shifts due to microgravity and their effects on bone: a review of current knowledge. Ann. Biomed. Eng..

[5] Bucaro MA (2004). Bone cell survival in microgravity: evidence that modeled microgravity increases osteoblast sensitivity to apoptogens. Ann. NY. Acad. Sci..

[6] Zwart SR (2007). Lower body negative pressure treadmill exercise as a countermeasure for bed rest-induced bone loss in female identical twins. Bone..

[7] Rittweger J (2005). Muscle atrophy and bone loss after 90 days’ bed rest and the effects of flywheel resistive exercise and pamidronate: results from the LTBR study. Bone..

[8] Gupta S, Vijayaraghavan S, Uzer G, Judex S (2012). Multiple exposures to unloading decrease bone’s responsivity but compound skeletal losses in C57BL/6 mice. Am. J. Physiol. Regul. Integr. Comp. Physiol..

[9] Halloran BP (1986). The role of 1,25-dihydroxyvitamin D in the inhibition of bone formation induced by skeletal unloading. Endocrinology..

[10] Vico L (1995). Bone changes in 6-mo-old rats after head-down suspension and a reambulation period. J. Appl. Physiol..

[11] Pardo SJ (2005). Simulated microgravity using the random positioning machine inhibits differentiation and alters gene expression profiles of 2T3 preosteoblasts. Am. J. Physiol. Regul. Integr. Comp. Physiol..

[12] Patel MJ (2007). Identification of mechanosensitive genes in osteoblasts by comparative microarray studies using the rotating wall vessel and the random positioning machine. J. Cell. Biochem..

[13] Borst AG, Loon J (2009). Technology and developments for the random positioning machine, RPM. Microgravity. Sci. Tec..

[14] Hoson T, Kamisaka S, Masuda Y, Yamashita M, Buchen B (1997). Evaluation of the three-dimensional clinostat as a simulator of weightlessness. Planta..

[15] Loon J (2007). Some history and use of the Random Positioning Machine, RPM, in gravity related research. Adv. Space Res..

[16] Wuest SL, Richard S, Kopp S, Grimm D, Egli M (2015). Simulated microgravity: critical review on the use of random positioning machines for mammalian cell culture. Biomed. Res. Int..

[17] Hu LF, Li JB, Qian AR, Wang F, Shang P (2015). Mineralization initiation of MC3T3-E1 preosteoblast is suppressed under simulated microgravity condition. Cell. Bio. Int..

[18] Ontiveros C, Mccabe LR (2003). Simulated microgravity suppresses osteoblast phenotype, Runx2 levels and AP-1 transactivation. J. Cell. Biochem..

[19] Zayzafoon M, Gathings WE, Mcdonald JM (2004). Modeled microgravity inhibits osteogenic differentiation of human mesenchymal stem cells and increases adipogenesis. Endocrinology.

[20] Peter S, Pedersen LB, Christensen ST (2010). The primary cilium at a glance. J. Cell. Sci..

[21] Xie YF (2016). Pulsed electromagnetic fields stimulate osteogenic differentiation and maturation of osteoblasts by upregulating the expression of BMPRII localized at the base of primary cilium. Bone..

[22] Schwartz EA, Leonard ML, Bizios R, Bowser SS (1997). Analysis and modeling of the primary cilium bending response to fluid shear. Am. J. Physiol..

[23] Hoey DA, Kelly DJ, Jacobs CR (2011). A role for the primary cilium in paracrine signaling between mechanically stimulated osteocytes and mesenchymal stem cells. Biochem Biophys Res Commun..

[24] Masyuk AI (2013). Ciliary subcellular localization of TGR5 determines the cholangiocyte functional response to bile acid signaling. Am. J.Physiol. Gastr. L..

[25] Kwon RY, Temiyasathit S, Tummala P, Quah CC, Jacobs CR (2010). Primary cilium-dependent mechanosensing is mediated by adenylyl cyclase 6 and cyclic AMP in bone cells. FASEB J..

[26] Praetorius HA, Spring KR (2001). Bending the MDCK cell primary cilium increases intracellular calcium. J. Membrane. Bio..

[27] Maria IF (2006). Oral-facial-digital type I protein is required for primary cilia formation and left-right axis specification. Nat. Genet..

[28] Vincent M (2011). Bardet-Biedl syndrome highlights the major role of the primary cilium in efficient water reabsorption. Kidney Int..

[29] Jagger D (2011). Alstrom Syndrome protein ALMS1 localizes to basal bodies of cochlear hair cells and regulates cilium-dependent planar cell polarity. Hum. Mol. Genet..

[30] Ou Y (2009). Adenylate cyclase regulates elongation of mammalian primary cilia. Exp. Cell. Res..

[31] Wilson NF, Iyer JK, Buchheim JA, Meek W (1998). Regulation of flagellar length in Chlamydomonas. Semin. Cell. Dev. Biol..

[32] Sharma N, Kosan ZA, Stallworth JE, Berbari NF, Yoder BK (2011). Soluble levels of cytosolic tubulin regulate ciliary length control. Mol. Biol. Cell..

[33] Kim J (2010). Functional genomic screen for modulators of ciliogenesis and cilium length. Nature..

[34] Palmer KJ, Maccarthy-Morrogh L, Smyllie N, Stephens DJ (2011). A role for Tctex-1 (DYNLT1) in controlling primary cilium length. Eur. J. Cell. Biol..

[35] Miyoshi K, Kasahara K, Miyazaki I, Asanuma M (2011). Factors that influence primary cilium length. Acta. Med. Okayama..

[36] Palmer KJ, Hughes H, Stephens DJ (2009). Specificity of cytoplasmic dynein subunits in discrete membrane-trafficking steps. Mol. Biol. Cell..

[37] Manzey D, Lorenz B (1999). Human performance during spaceflight. Hum. Perf. Extrem. Environ..

[38] Kozlovskaya IB, Grigoriev AI (2004). Russian system of countermeasures on board of the International Space Station (ISS): the first results. Acta Astronautica..

[39] Mamta PN, Diana R (2013). The current state of bone loss research: data from spaceflight and microgravity simulators. J. Cell. Biochem..

[40] Jee WS, Wronski TJ, Morey ER, Kimmel DB (1983). Effects of spaceflight on trabecular bone in rats. Am. J. Physiol..

[41] Turner RT, Evans GL, Wakley GK (1995). Spaceflight results in depressed cancellous bone formation in rat humeri. Aviat Space Environ. Med..

[42] Borer DT (2005). Physical activity in the prevention and amelioration of osteoporosis in women. Sports Med..

[43] Hoey DA, Tormey S, Ramcharan S, O’Brien FJ, Jacobs CR (2012). Primary cilia-mediated mechanotransduction in human mesenchymal stem cells. Stem Cells..

[44] Yan JL (2015). Pulsed electromagnetic fields promote osteoblast mineralization and maturation needing the existence of primary cilia. Mol. Cell. Endocrinol..

[45] Delaine-Smith RM, Sittichokechaiwut A, Reilly GC (2014). Primary cilia respond to fluid shear stress and mediate flow-induced calcium deposition in osteoblasts. FASEB J..

[46] Gershovich PM, Gershovich IG, Buravkova LB (2009). Cytoskeleton structures and adhesion properties of human stromal precursors under conditions of simulated microgravity. Tsitologiia..

[47] Bershteyn M, Atwood SX, Woo WM, Li M, Oro AE (2010). MIM and cortactin antagonism regulates ciliogenesis and hedgehog signaling. Dev. Cell..

[48] Sen B (2015). Intranuclear actin regulates osteogenesis. Stem Cells..

[49] Dai Z (2011). Altered actin dynamics and functions of osteoblast-like cells in parabolic flight may involve ERK1/2. Microgravity. Sci. Tec..

[50] Espinha LC, Hoey DA, Fernandes PR, Rodrigues HC, Jacobs CR (2014). Oscillatory fluid flow influences primary cilia and microtubule mechanics. Cytoskeleton..

