# Supplementary material for: Microgravity induces inhibition of osteoblastic differentiation and mineralization through abrogating primary cilia
Source: Sci Rep. 2017 May 12;7:1866. doi: 10.1038/s41598-017-02049-9 (PMC5431935; doi:10.1038/s41598-017-02049-9)
Supplement: Supplementary file 1 — Supplementary information [file 41598_2017_2049_MOESM1_ESM.pdf]

Microgravity induces inhibition of osteoblastic differentiation and mineralization through abrogating primary cilia

Wengui Shi (PhD)[1,2], Yanfang Xie (MSc)[3], Jinpeng He (PhD)[1], Jian Zhou (PhD)[3], Yuhai Gao (MSc)[3], Wenjun Wei (PhD)[1,2], Nan Ding (PhD)[1], Huiping Ma (PhD)[3], Cory J Xian (PhD)[4], Keming Chen (PhD)[3*], Jufang Wang (PhD)[4*]

[1] Gansu Key laboratory of Space Radiobiology, Institute of Modern Physics, Chinese Academy of Sciences, Lanzhou 730000, P.R China;

[2] University of Chinese Academy of Sciences, Beijing 100049, P.R China;

[3] Institute of Orthopaedics, Lanzhou General Hospital, Lanzhou Command of CPLA, Lanzhou 730050, P.R China;

[4] Sansom Institute for Health Research, School of Pharmacy and Medical Sciences, University of South Australia, Adelaide, SA 5001, Australia

*Correspondence and requests for materials should be addressed to J.F.W. (email: jufangwang@impcas.ac.cn) or K.M.C. (email: chenkm@lut.cn)

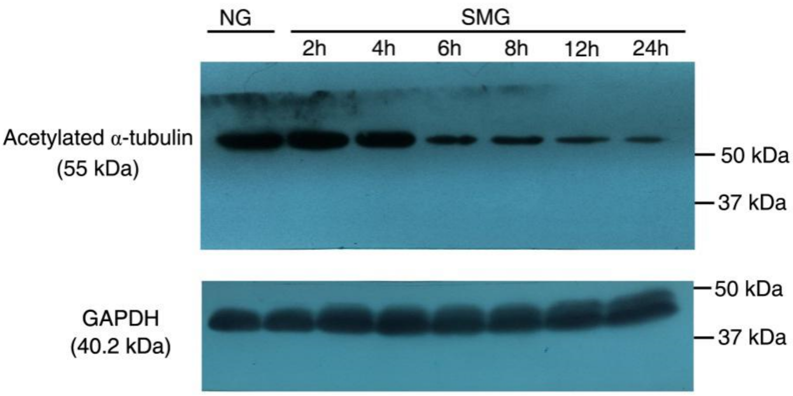

Supplementary Figure 1. A typical full-length Western blot of acetylated α-tubulin in rat calvarial osteoblasts (ROBs). The protein expression levels of acetylated α-tubulin (55 kDa) in ROBs with or without random positioning machine (RPM) treatment. GAPDH (40.2 kDa) was used as an internal control.

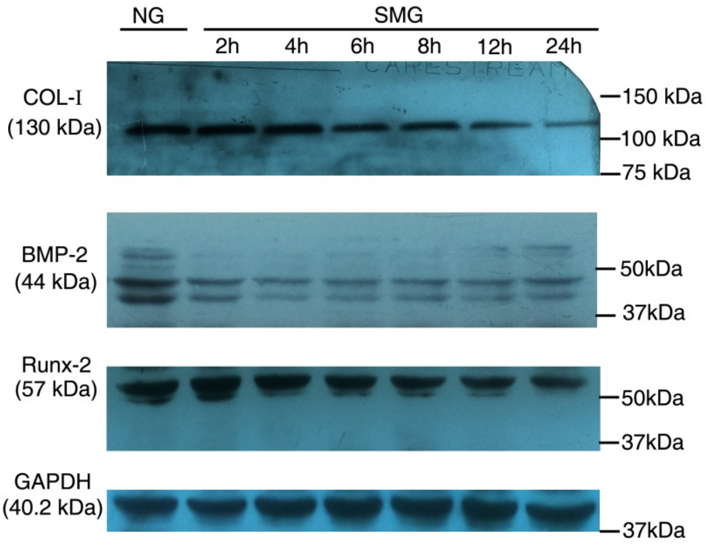

Supplementary Figure 2. Typical full-length Western blots of COL-I, BMP-2 and Runx-2 in rat calvarial osteoblasts (ROBs). The protein expression levels of COL-I (130 kDa), BMP-2 (44 kDa) and Runx-2 (57 kDa) in ROBs with or without random positioning machine (RPM) treatment. GAPDH (40.2 kDa) was used as an internal control. The blot of BMP-2 showed two bands, which probably was due to some non-specific binding, and the correct

band of 44 kDa was used for the final results.

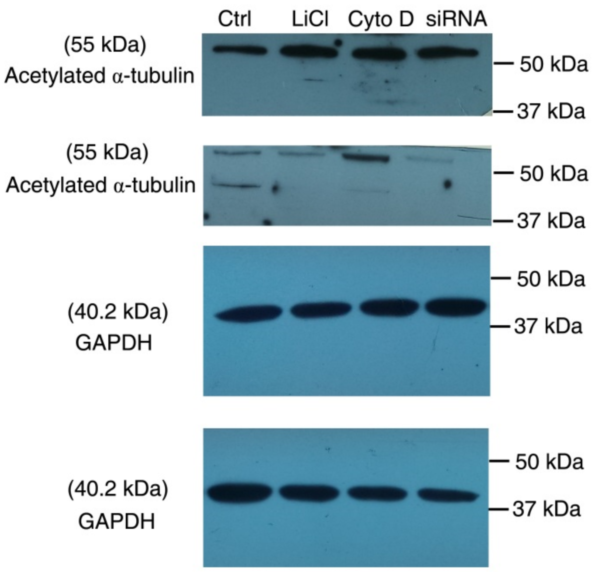

Supplementary Figure 3. Typical full-length Western blots of acetylated α-tubulin in rat calvarial osteoblasts (ROBs). The protein expression levels of acetylated α-tubulin in ROBs treated with LiCl, Cyto D or Dynlt1 siRNA. GAPDH (40.2 kDa) was used as an internal control.

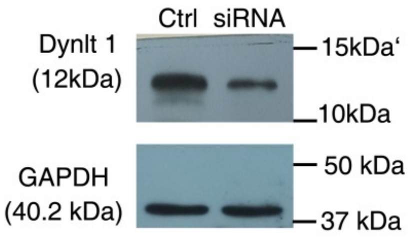

Supplementary Figure 4. A typical full-length Western blot of Dynlt1 in rat calvarial osteoblasts (ROBs). The protein expression levels of Dynlt1 (12kDa) in ROBs treated with control siRNA or Dynlt1 siRNA. GAPDH (40.2 kDa) was used as an internal control.

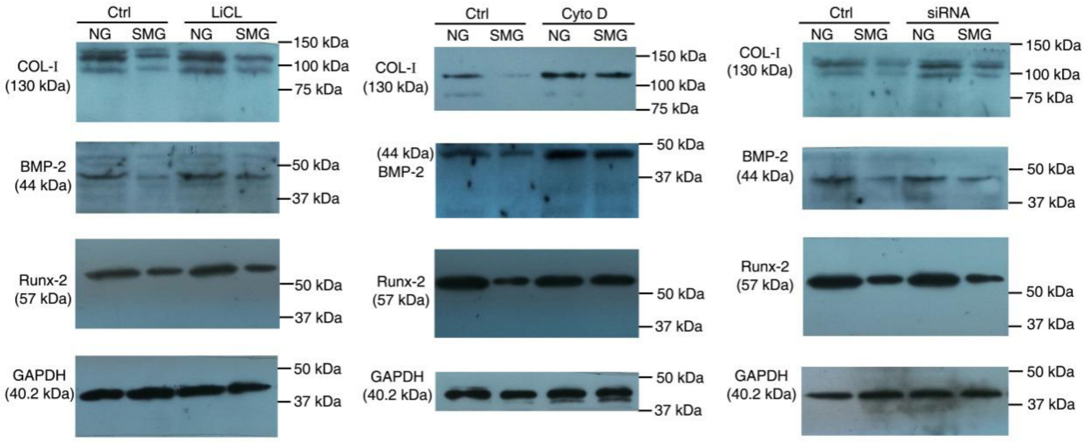

Supplementary Figure 5. Typical full-length Western blots of COL-I, BMP-2 and Runx-2 in rat calvarial osteoblasts (ROBs). The protein expression levels of COL-I (130 kDa), BMP-2 (44 kDa) and Runx-2 (57 kDa) in ROBs treated with LiCl, Cyto D or Dynlt1 siRNA. GAPDH (40.2 kDa) was used as an internal control. The blot of COL-I had two bands, which probably due to some non-specific binding, and the correct band of 130 kDa was used as the final result.

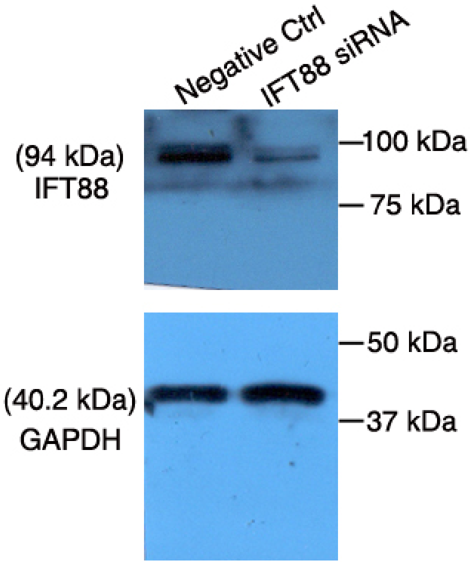

Supplementary Figure 6. A typical full-length Western blot of IFT88 in rat calvarial osteoblasts (ROBs). The protein expression levels of IFT88 (94kDa) in ROBs treated with control siRNA or IFT88 siRNA. GAPDH (40.2 kDa) was used as an internal control.

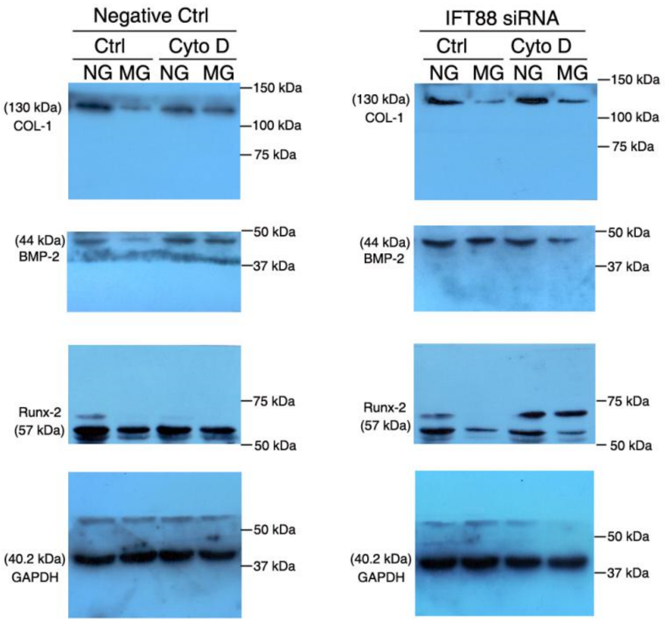

Supplementary Figure 7. Typical full-length Western blots of COL-I, BMP-2 and Runx-2 in rat calvarial osteoblasts (ROBs). The protein expression levels of COL-I (130 kDa), BMP-2 (44 kDa) and Runx-2 (57 kDa) in ROBs treated with LiCl, Cyto D or Dynlt1 siRNA. GAPDH (40.2 kDa) was used as an internal control.
